# Comparative Genomics Reveals Insight into Virulence Strategies of Plant Pathogenic Oomycetes

**DOI:** 10.1371/journal.pone.0075072

**Published:** 2013-10-04

**Authors:** Bishwo N. Adhikari, John P. Hamilton, Marcelo M. Zerillo, Ned Tisserat, C. André Lévesque, C. Robin Buell

**Affiliations:** 1 Department of Plant Biology, Michigan State University, East Lansing, Michigan, United States of America; 2 Department of Bioagricultural Sciences and Pest Management Colorado State University, Fort Collins, Colorado, United States of America; 3 Agriculture and Agri-Food Canada, Ottawa, Ontario, Canada and Department of Biology, Carleton University, Ottawa, Ontario, Canada; Virginia Tech, United States of America

## Abstract

The kingdom Stramenopile includes diatoms, brown algae, and oomycetes. Plant pathogenic oomycetes, including *Phytophthora*, *Pythium* and downy mildew species, cause devastating diseases on a wide range of host species and have a significant impact on agriculture. Here, we report comparative analyses on the genomes of thirteen straminipilous species, including eleven plant pathogenic oomycetes, to explore common features linked to their pathogenic lifestyle. We report the sequencing, assembly, and annotation of six *Pythium* genomes and comparison with other stramenopiles including photosynthetic diatoms, and other plant pathogenic oomycetes such as *Phytophthora* species, *Hyaloperonospora arabidopsidis,* and *Pythium ultimum* var. *ultimum*. Novel features of the oomycete genomes include an expansion of genes encoding secreted effectors and plant cell wall degrading enzymes in *Phytophthora* species and an over-representation of genes involved in proteolytic degradation and signal transduction in *Pythium* species. A complete lack of classical RxLR effectors was observed in the seven surveyed *Pythium* genomes along with an overall reduction of pathogenesis-related gene families in *H. arabidopsidis*. Comparative analyses revealed fewer genes encoding enzymes involved in carbohydrate metabolism in *Pythium* species and *H. arabidopsidis* as compared to *Phytophthora* species, suggesting variation in virulence mechanisms within plant pathogenic oomycete species. Shared features between the oomycetes and diatoms revealed common mechanisms of intracellular signaling and transportation. Our analyses demonstrate the value of comparative genome analyses for exploring the evolution of pathogenesis and survival mechanisms in the oomycetes. The comparative analyses of seven *Pythium* species with the closely related oomycetes, *Phytophthora* species and *H. arabidopsidis*, and distantly related diatoms provide insight into genes that underlie virulence.

## Introduction

Oomycetes are a diverse group of organisms that morphologically resemble Fungi, yet are members of the *Straminipila* ( = stramenopile) and are more closely related to organisms in aquatic environments such as brown algae and diatoms. There is continued discussion on the higher level nomenclature of *Straminipila* within the kingdom *Chromista* [1], [2], [3] which when united with the alveolates, comprise the chromalveolate superkingdom [4], [5]. The algal stramenopiles are secondarily photosynthetic, however, non-photosynthetic stramenopiles, such as the oomycetes, share numerous genes of putative phototrophic origin [6], [7] lending support to the hypothesis that the straminipilous ancestor was photosynthetic [5]. Nevertheless, there has been continuous debate on the existence of chromalveolate hypothesis and the photosynthetic origin of the stramenopiles [8]. The oomycetes include a diverse range of free-living water molds and pathogens of plants, mammals, insects, fish, crustaceans, algae, and various microbes, including fungi [9], [10], [11], [12], [13]. Plant pathogenic oomycetes cause devastating diseases of crop, ornamental, and native species and are thought to not only be the most important group of pathogens of dicotyledonous plants [14] but also often the source of yield reduction in cereal crop species [15], [16], [17]. Some of the most damaging oomycete genera are *Aphanomyces* [18], *Peronospora* [19], *Phytophthora* [20], *Plasmopara* [21], *Pseudoperonospora* [22], and *Pythium* [23] species; the wide host range of these genera, coupled with the diversity of diseases they cause, pose a challenge to the development of durable disease control strategies in plants.

**Figure 1:**
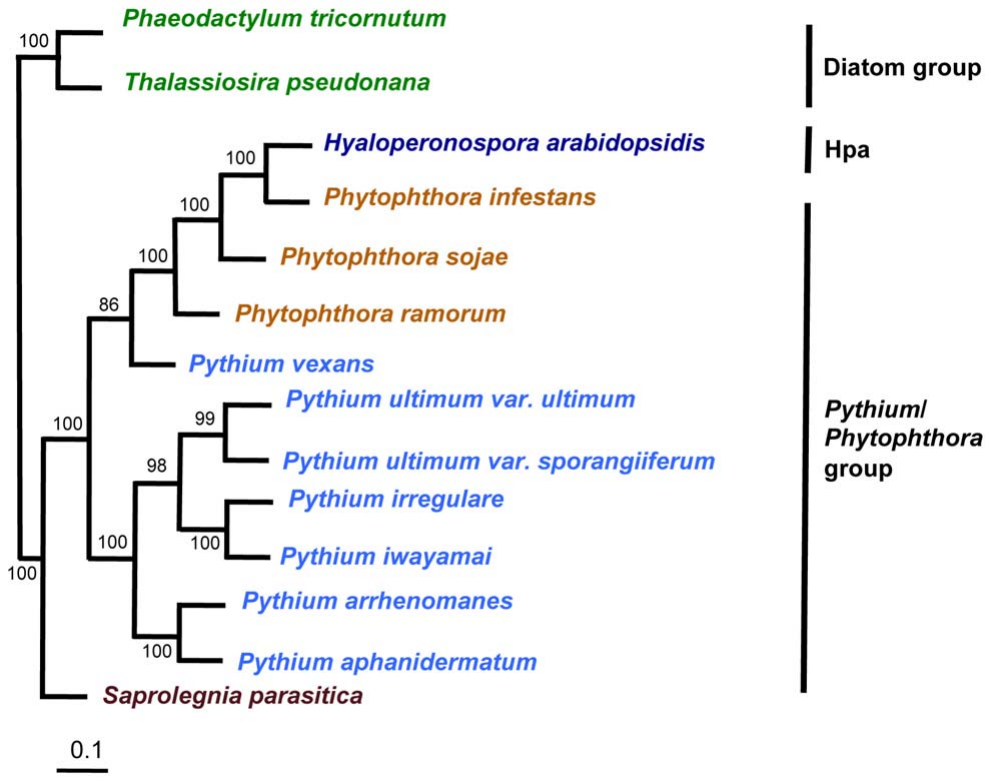
Phylogeny of oomycetes. Phylogeny of the large rDNA subunit of select oomycetes as inferred by Bayesian analysis. The phylogenetic tree was constructed using rDNA sequences from 14 stramenopiles. Numbers on the branches are Bayesian posterior probability values calculated using MrBayes [110]. Hpa, *Hyaloperonospora arabidopsidis*.

Within the oomycetes, *Pythium* species belongs to the peronosporalean lineage that includes hemibiotrophic *Phytophthora* species and the obligate biotrophic *Hyaloperonospora* species (Figure 1). The genus *Pythium* comprises more than 250 described species with 50% of these accepted by the community and currently classified into 11 phylogenetic clades [23]. Recently, one of these clades was shown to be closer to *Phytophthora* and the new genus *Phytopythium* has been described but official renaming of all *Pythium* species in clade K has not yet occurred [24], [25]. Most *Pythium* species are saprobes or facultative plant pathogens causing a wide variety of diseases, including seed rots and damping-off, root, stem and fruit rots, foliar blights, and postharvest decay [26], [27], [28], [29]. Some *Pythium* species have been reported to be parasitic to fungi and a few have been evaluated for biological control against other oomycete plant pathogens [30], [31], [32]. Some *Pythium* species are parasites of insects [12], fish [13], algae [33] and at least one species, *Pythium insidiosum*, infects mammals including humans [11]. Members of the genus *Pythium* differ from other oomycetes, including *Phytophthora* species, in morphology, genetic features [13], [14], and lifestyle. *Pythium* species are primarily necrotrophs and their sporangia produce a vesicle prior to the differentiation and release of zoospores whereas *Phytophthora* species are hemibiotrophs with zoospore differentiation occurring directly within the sporangia [34].

*Pythium* species are genetically diverse [35] and exhibit significant variation in terms of virulence, host range, and distribution [10], [13]. Despite being members of the *Pythium* lineage that produces filamentous sporangia, *Pythium aphanidermatum* and *Pythium arrhenomanes* have distinct temperature optima and levels of virulence [36]. Similar to *Pythium ultimum* var. *ultimum, Pythium aphanidermatum* has a broad host range and is frequently found in greenhouses and high temperature conditions [10], [13], [37], [38]. This contrasts with *Pythium arrhenomanes* which is more restricted in host range with a preference for monocotyledonous plants [10]. Similar to *Py. ultimum* var. *ultimum*, *Pythium irregulare* is a species with globose sporangia, highly virulent at cooler temperatures [10], occurs in a broad eco-geographic range, and exhibits high genetic and morphological diversity [23], [39], [40], [41]. Unlike other species, *Pythium vexans*, which belongs to clade K and should be renamed as *Phytopythium*, causes root rot disease in many economically important tropical trees such as durian and rubber plants [42], [43]. *Py. vexans* also belongs to a species that has a wide range of genetic variation [44]. *Py. ultimum* var. *sporangiiferum* is in the *P. ultimum* species complex which has a wide genetic variation. In this study, we treat *Py. ultimum* var. *sporangiiferum* as a separate species because there is no evidence of gene flow between the two *Py. ultimum* varieties using a large collection of geographically overlapping strains from each group [45]. *Pythium iwayamai* is pathogenic to monocot grasses, grows at temperatures as low as 10°C, and causes snow rot disease in economically important crops such as turfgrass, barley, and winter wheat [13], [46], [47], [48]. The diversity in host range and optimal environmental conditions for infection makes the genus *Pythium* a good model to study plant-necrotroph interactions and to identify genes involved in interspecific variation in pathogenicity.

The development of second generation sequencing platforms [49], [50] offers an opportunity to sequence and perform comparative analyses of a large number of genomes, including phytopathogens [51]. A number of genome sequences of plant pathogenic oomycete are now available, including the downy mildew pathogen *Hyaloperonospora arabidopsidis* [52], three *Phytophthora* species (*Ph. infestans* [6], *Ph. ramorum*, *Ph. sojae* [7]), and *Py. ultimum* var. *ultimum* [53]. To date, comparative analyses of oomycete pathogens have shown variation in genome size, genome content, and evolution of host-pathogen interactions [7], [52], [53], [54], [55], [56]. For example, several gene families that facilitate the infection process are expanded [57] in *Phytophthora* species and significantly reduced in *Py. ultimum* var. *ultimum* and *H. arabidopsidis* [52], [53]. The availability of genome sequences of two species of diatoms, *Phaeodactylum tricornutum* [57], and *Thalassiosira pseudonana* [58], permits comparative analyses within the stramenopiles with respect to evolution of pathogenicity. Here, we describe the genome sequence assemblies and annotation for six additional *Pythium* species and identify genes involved in pathogenicity and necrotrophic lifestyle. Comparative analyses of seven *Pythium* species with closely related oomycetes, three *Phytophthora* species, *H. arabidopsidis*, and distantly related autotrophic diatoms provided insight into genes that underlie pathogenicity.

## Results and Discussion

### Genome Sequencing, Assembly, and Annotation

The sequences of six *Pythium* genomes (*Py. aphanidermatum* (DAOM BR444 = CBS 132490), *Py. arrhenomanes* (ATCC 12531 = CBS 324.62), *Py. irregulare* (DAOM BR486 = CBS 250.28), *Py. iwayamai* (DAOM 242034 = CBS 132417), *Py. ultimum* var. *sporangiiferum* (DAOM BR650 = CBS 219.65), and *Py. vexans* (DAOM BR484 = CBS 119.80); Table 1) that provide a broad representation of the genus *Pythium* (Figure 1) were generated using pyrosequencing with the Roche 454 or the Illumina Genome Analyzer (GA) II sequencing-by-synthesis platform. For the five *Pythium* species (*Py. arrhenomanes*, *Py. irregulare*, *Py. iwayamai*, *Py. ultimum* var. *sporangiiferum*, and *Py. vexans*), 6.6 to 14.4 Gb of purity-filtered (PF) reads were generated by the Illumina GAII/IIx sequencer (Table S1) while for *Py. aphanidermatum,* 507 Mb of single-end and 137.5 Mb of paired-end reads were generated using pyrosequencing (see Methods). Assembled genomes of six *Pythium* species yielded 3,685 to 11,542 contigs with an N_50_ contig length ranging from 9.8 to 37.4 Kb (Table 2, Table S2, Table S3). The total contig length/genome size ranged from 33.9 to 44.7 Mb in the six *Pythium* species, comparable to 42.8 Mb in *Py. ultimum* var. *ultimum* that was sequenced previously using Sanger-based methods [53]. The maximum contig length of the six *Pythium* species was comparable to *Py. ultimum* var. *ultimum* [53], ranging from 96.3 to 222.5 Kb (Table S3). In general, our study shows that *Pythium* species have smaller genomes than the three *Phytophthora* species or *H. arabidopsidis*.

**Table 1:** Species name, accession numbers, host/substrate and geographical origin of the *Pythium* strains sequenced in this study.

Species	CBS accession¶	Other accession identifiers	Host/substrate	Origin
*Py. aphanidermatum*	132490	DAOM BR444	*Cucumis sativus*	BC, Canada
*Py. arrhenomanes*	324.62	ATCC 12531	*Zea mays*	WI, USA
*Py. irregulare*	250.28	DAOM BR486	*Phaseolus vulgaris*	Netherlands
*Py. iwayamai*	132417	DAOM 242034	*Poa annua*	CO, USA
*Py. ultimum* var. *sporangiiferum*	219.65	DAOM BR650	*Chenopodium album*	USA
*Py. vexans*	119.80	DAOM BR484	Soil	Iran

¶ CBS: CBS-KNAW fungal biodiversity center, Utrecht, Netherlands (http://www.cbs.knaw.nl); DAOM: Culture collection of Agriculture and Agri-Food Canada, Ottawa, Canada; ATCC: American Type Culture Collection, Manassas, VA, USA.

**Table 2:** Assembly and annotation statistics† of thirteen stramenopiles.

Species	Number ofcontigs	N50 contiglength (Kb)	Total contig length/genome size (Mb) ¶	Number ofgenes	Number oftranscripts
This study					
*Py. aphanidermatum*	5,667	37.39	35.9	12,305	12,312
*Py. arrhenomanes*	10,978	9.77	44.7	13,805	13,805
*Py. irregulare*	5,887	23.22	42.9	13,804	13,805
*Py. iwayamai*	11,542	11.01	43.3	14,875	14,875
*Py. ultimum* var. *sporangiiferum*	5,482	19.11	37.7	14,086	14,096
*Py. vexans*	3,685	29.24	33.9	11,957	11,958
Published genomes					
*H. arabidopsidis* †	3,138*	NA	100	14,543	NA
*Ph. infestans* †	4,921	44.5	240	17,797	NA
*Ph. ramorum* †	7,588	47.5	65	15,743	NA
*Ph. sojae* †	5,577	105.7	95	19,027	NA
*P. tricornutum* †	NA	NA	27.4	10,402	NA
*Py. ultimum* var. *ultimum* †	1,747*	124	42.8	15,291	15,323
*T. pseudonana* †	NA	NA	32.4	11,776	NA

For more detailed statistics see Table S2 and Table S3. NA, not available.

¶ Total contig length/total genome size.

† Statistics obtained from published genome datasets [6], [7], [52], [53], [57], [58].

* Number of scaffolds.

Annotation of the six *Pythium* species revealed 11,957 to 14,875 predicted genes, comparable with the 15,291 genes annotated in *Py. ultimum* var *ultimum* [53]. Overall, the number of genes in any *Pythium* species is less than in *Phytophthora* species (15,743 to 19,027 genes) [6], [7] yet similar to *H. arabidopsidis* (14,543 genes) [52]. Average gene length was similar among all *Pythium* species, ranging from 1,495 to 1,767 bp (Table S3). Analysis of the intron/exon structure showed that the majority of *Pythium* species have 1.5 to 1.7 introns per gene, consistent with that of *Py. ultimum* var. *ultimum* (1.6 introns per gene) and *Ph. infestans* (1.7 introns per gene). Average exon length in the six newly sequenced *Pythium* genomes ranged from 312 to 630 bp, consistent with *Py. ultimum* var. *ultimum* (498 bp). Similar to *Py. ultimum* var. *ultimum* [53], 58 to 65% of all predicted genes from six *Pythium* species contain an InterPro protein domain, comparable to that observed with *Phytophthora* species (55 to 66%) [6], [7].

To aid in our genome annotation, we performed whole transcriptome sequencing (RNA-sequencing (RNA-seq)) of *Py. arrhenomanes*, *Py. irregulare*, *Py. iwayamai*, and *Py. vexans*. For these four species, a single pooled cDNA library was constructed for each species using RNA isolated from mycelium grown under five different growth conditions (nutrient-rich medium, nutrient-starved condition, fungicide treatment, as well as heat and cold temperature stress) and sequenced using the Illumina GAII. The total number of purity filtered reads ranged from 21.7 to 31.7 million reads per library with 82–88% of reads mapping to the cognate genome (Table S4) indicating a similar performance of library construction and sequencing across the samples. The minimum fragments per kilobase of exon model per million mapped fragments (FPKM) value for all growth conditions was 0 while the maximum FPKM ranged from 10,890 for sylvaticin, an elicitin-like protein in *Py. arrhenomanes*, to 30,906 for the INF1 elicitin in *Py. vexans*. The percentage of genes with transcript support ranged from 71% in *Py. iwayamai* to 81% in *Py. vexans* (Table S4). A gene was considered expressed if the FPKM value and FPKM 95% confidence interval lower boundary was greater than 0.001 and zero, respectively.

### Core and Species-specific Genes and Gene Families in *Pythium*

**Figure 2:**
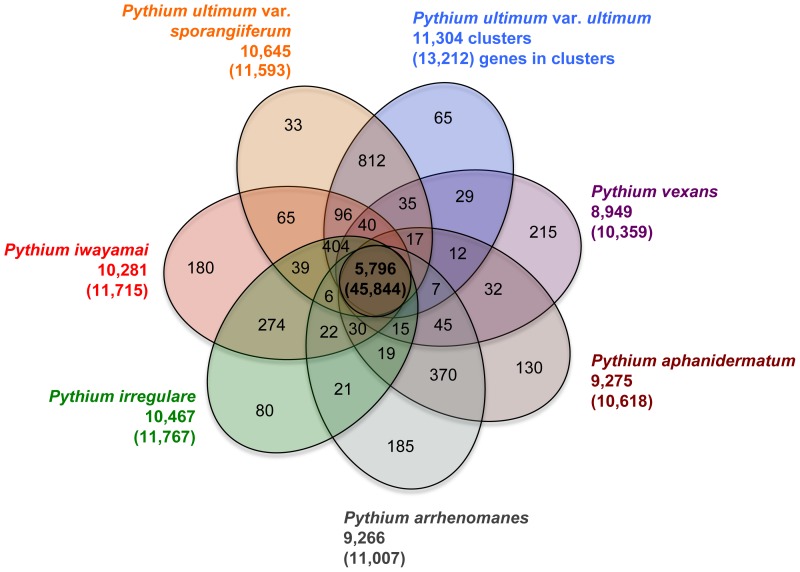
Gene families shared by *Pythium* species. The predicted proteomes of the seven *Pythium* species were clustered using OrthoMCL [59] to identify orthologs and close paralogs. The number of gene families shared between the species and total number of clustered genes (numbers in parentheses) are indicated. The numbers outside the Venn diagram show the total number of orthologous clusters and number of genes (in parentheses) within those clusters for each species. Pap, *Pythium aphanidermatum;* Par, *Pythium arrhenomanes*; Pir, *Pythium irregulare*; Piw, *Pythium iwayamai*; Puls, *Pythium ultimum* var. *sporangiiferum*; Pult, *Pythium ultimum* var. *ultimum*; Pve, *Pythium vexans.*

To identify the core *Pythium* proteome, we clustered orthologs and close paralogs in seven predicted *Pythium* proteomes (the newly sequenced six *Pythium* species and the previously sequenced *Py. ultimum* var. *ultimum* [53]) using OrthoMCL [59]. Of the 95,668 protein-coding genes, 80,271 genes clustered into 13,803 gene families with 15,397 genes as singletons. A total of 45,844 genes, clustered into 5,796 gene families, were common to all *Pythium* species, hereafter referred to as the core *Pythium* proteome (Figure 2). A total of 888 gene families containing 2,233 genes were unique to each species, ranging from 33 gene families in *Py. ultimum* var. *sporangiiferum* to 215 gene families in *Py. vexans*.

To gain insight into the unique features of the core *Pythium* genes, we compared the frequency of occurrence of protein family domains in the core *Pythium* gene family set and the species-specific genes (clustered genes and singletons). First, comparisons were made between core *Pythium* genes and the rest of the genes from each species. The core *Pythium* genes were enriched in genes involved in pathogenesis and signaling processes including elicitin (IPR002200), necrosis-inducing (IPR008701), peptidase C1A (IPR000668), protease inhibitor (Kazal-type (IPR011497)), and HECT ubiquitin ligase (IPR000569) whereas genes involved in transport activities (IPR001140, IPR018108) are underrepresented (P<0.05) (Table S5).

Second, the species-specific genes were compared to the rest of the genes from those particular species. Several transporter- (ABC (IPR001140) and nucleotide-sugar transporter-related (IPR007271)) domains were highly over-represented in *Py. vexans*-specific genes (Chi-square test with Bonferroni correction, *P*<0.001). The *Py. aphanidermatum*-specific genes were highly enriched (*P*<0.01) for aspartic peptidase (IPR021109), endoglucanase (IPR009009), cutinase (IPR000675), and pectate lyase (IPR002022) domains. The highly represented domains in *Py. arrhenomanes* were protease inhibitor (Kazal-type (IPR011497)), cutinase (IPR000675), necrosis inducing (IPR008701), and pectate lyase (IPR002022). Similarly, pathogenesis related domains such as peptidase (IPR006026) and proteinase inhibitor I25 (cystatin (IPR018073)) were highly represented (*P*<0.05) in *Py. irregulare*-specific genes. The leucine-rich repeat (LRR) containing domain (IPR001611), carbonic anhydrase (IPR018338), and chitinase II (IPR011583) were over-represented (*P*<0.05) in *Py. iwayamai*-specific genes (Table S5). A number of these protein domains have been shown to be implicated in plant-pathogen interaction in different oomycete pathogens [55], [60]. In general, we observed higher representation of protein domains potentially involved in degradation of host tissues (e.g., glycoside hydrolase) and establishment of infection structure (e.g., elicitin, necrosis inducing proteins, protease inhibitors) in the core *Pythium* gene set leading to necrotrophic life style.

### Metabolism of Complex Carbohydrates

Carbohydrate-active enzymes (CAZymes) are involved in the biosynthesis and degradation of diverse glycoconjugates, oligosaccharides, and polysaccharides [61] and have a central role in the breakdown of the plant cell wall by plant pathogens thereby serving as pathogenicity factors. These enzymes can also be involved in the biosynthesis, breakdown, and modification of the oomycete cell wall and structural polysaccharides as part of growth and development. Thus, comparison of the CAZyme content would provide insights into metabolic and enzymatic diversity in oomycete pathogens. Putative CAZymes in *Pythium* species were identified using the CAZymes Analysis Toolkit (CAT) [62] and correspondence between CAZyme families and protein family domains was analyzed. The comparison of the glycoside hydrolase (GH), glycosyltransferases (GTs), polysaccharide lyase (PL), and carbohydrate esterase (CE) in the *Pythium* genomes revealed that these organisms exhibit substantial variation in number of CAZymes (Table S6). The CE and carbohydrate-binding module (CBM) classes were poorly represented in all *Pythium* genomes.

**Figure 3:**
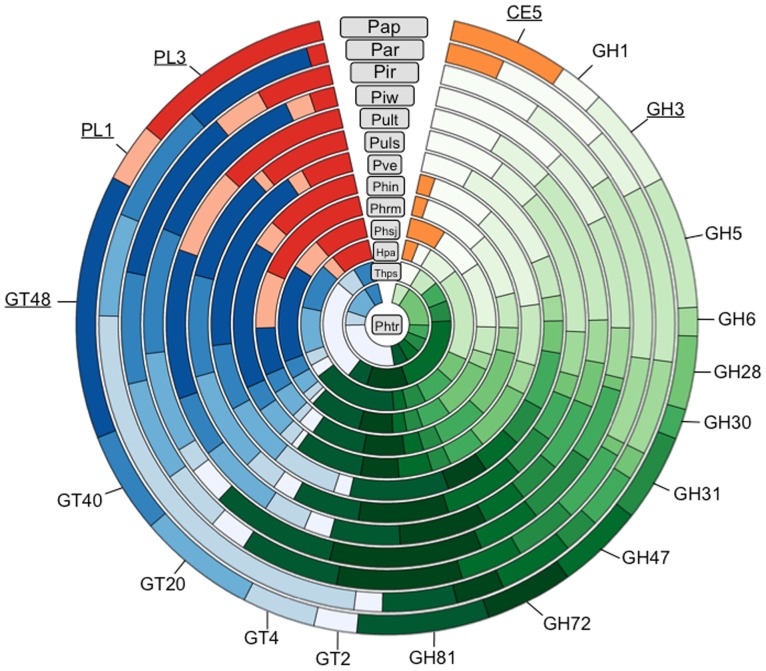
Distribution of various carbohydrate-active enzymes (CAZymes) in stramenopile genomes. The CAZymes coding genes were annotated using the CAZymes Analysis Toolkit- CAT [62] according to the CAZy database [61] in combination with protein family domain analyses. Gene families absent in at least 2 species are underlined. Comparison of total CAZymes from different classes is listed in Table S6. CE, carbohydrate esterase; GH, glycoside hydrolase; GT, glycosyl transferase; PL, polysaccharide lyase; Pap, *Pythium aphanidermatum*; Par, *Pythium arrhenomanes*; Pir, *Pythium irregulare*; Piw, *Pythium iwayamai*; Pult, *Pythium ultimum* var. *ultimum*; Puls, *Pythium ultimum* var. *sporangiiferum*; Pve, *Pythium vexans*; Phin, *Phytophthora infestans*; Phrm, *Phytophthora ramorum*; Phsj, *Phytophthora sojae*; Hpa, *Hyaloperonospora arabidopsidis*; Thps, *Thalassiosira pseudonana*; Phtr, *Phaeodactylum tricornutum*.

Interestingly, we identified eight and six cutinase-encoding genes (CE5 family) in *Py. aphanidermatum* and *Py. arrhenomanes*, respectively, but not in the other *Pythium* genomes (Figure 3, Table S6, Table S7) suggesting that the evolution of these phytopathogens led to different degrees of reduction in their cutin degrading capabilities. *Pythium* species have a relatively smaller set of GH-encoding genes (77 to 114 members) compared to all *Phytophthora* species (166–216 members) yet strikingly larger than the repertoire of the biotroph *H. arabidopsidis* (72 members) and the diatoms (31–32 members) (Table S6) in agreement with previous findings [52], [53], [56]. The GH superfamily was the most highly represented CAZyme superfamily in all *Pythium* genomes with PL the least represented (3 families). We observed that in general *Pythium* species have a highly reduced set of secreted CAZymes when compared to *Phytophthora* species, which underwent gene expansion [63]. The differential ability of oomycete pathogens to produce different hydrolytic enzymes acting on different complex carbohydrate molecules could determine their infection strategy, host range, and most likely contribute to the different virulence mechanisms between oomycete pathogens. An in-depth study of the *Pythium*-CAZymes is reported in a companion paper (Zerillo *et al.* PLoS One, this issue).

### The *Pythium* Secretome

*Pythium* species, like many oomycete pathogens, secrete effector proteins as well as degradative enzymes that alter host physiology and facilitate colonization. Indeed, the genomes of *Ph. infestans, Ph. ramorum, Ph. sojae*, *Py. ultimum* var. *ultimum,* and *H. arabidopsidis* contain large complex families of effector genes that encode secreted proteins which have been implicated in pathogenesis [6], [7], [52], [53]. Secreted proteins in the seven *Pythium* proteomes were predicted using SignalP v3.0 [64] and transmembrane domains predicted with TMHMM [65]. In total, 834 to 1,008 proteins were predicted to be secreted (using the criteria described in Materials and Methods) in the seven *Pythium* species (Table S8). Genes encoding the predicted secreted proteins were then clustered using OrthoMCL revealing 1,086 clusters containing 4,921 secreted proteins while 1,592 were singletons. A total of 76 clusters containing 782 secreted proteins were common to all *Pythium* species, hereafter referred to as core *Pythium* secretome (Figure S1). Of the total, 745 clusters have secreted proteins from at least three different species. The largest secretome gene family contains 25 members from all *Pythium* species, and encodes a polysaccharide lyase involved in host cell wall degradation. Other families of secreted core proteins in *Pythium* include elicitins, protease inhibitors, cellulose-binding elicitor lectin (CBEL)-like proteins with CBM, and expanded families of cell wall degrading enzymes. Overall, depending on the species, 63–78% of the predicted secreted proteins in *Pythium* species surveyed have expression support (Table S8).

To augment our functional annotation, we annotated the predicted secretome for PFAM domains using InterProScan [66] and associated Gene Ontology (GO) [67] terms using Blast2GO [68]. We also compared the frequency of these annotations with the non-secreted proteome using Chi-square tests with Bonferroni correction [69]. Pathogenesis (GO:0009405), proteolysis (GO:0006508), and carbohydrate metabolic processes (GO:0005975) were highly enriched in the core *Pythium* secretome (*P*<0.001) relative to the rest of the predicted proteome from *Pythium* (Table S9). The protein domains showing the highest enrichment in the core *Pythium* secretome are elicitin (IPR002200), glycoside hydrolases (IPR000322), glycosyl transferase (IPR001296), peptidase C1A (IPR000668), EGF-like domain (IPR006209), and pectate lyase (IPR004898) (*P*<0.001) (Table S10).

To document the protein family domains and biological functions enriched in the *Pythium* species-specific secretome, we compared the frequency of occurrence of PFAM domains and GO terms in the species-specific secretomes relative to the rest of the proteome from that particular species using Chi-square tests. The *Py. aphanidermatum*-specific secretomes were highly enriched for hydrolase activity (GO:0004553) including cutin hydrolase activity (GO:0050525) and carbohydrate metabolic process (GO:0005975) (*P*<0.05) (Table S9). Similarly, *Py. arrhenomanes*-specific secretomes were highly enriched for cellulose catabolic process (GO:0030245), hydrolase activity (GO:0004553), and pectin lyase activity (GO:0047490). The *Py. iwayamai*-specific secretomes were highly enriched for peptidase activity (GO:0008233), transmembrane transport (GO:0055085), and nucleic acid binding (GO:0003676). The most represented GO terms in *Py. ultimum* var. *sporangiiferum*-specific secretome were cysteine-type peptidase activity (GO:0008234), cellulose binding (GO:0030248), and isomerase activity (GO:0016853). The transmembrane transport (GO:0055085) as well as sugar binding and sugar modification activities (GO:0005529, GO:0016787, GO:0004650) were most enriched in *Py. vexans*-specific secretome while pectate lyase (GO:0030570), proteolysis (GO0006508), and glycosyl bonds hydrolase activity (GO:0016798) were the most enriched GO terms in the *Py. irregulare*-specific secretome. Enrichment of hydrolase, pectate lyase activity and cell wall modification process in species-specific secretome indicates that degrading host cell wall is one of the major functions of *Pythium* secretome as illustrated for other oomycete pathogens [55], [60].

Similarly, the protein family domains including cutinase (IPR000675) and glycoside hydrolase (IPR000743) that hydrolyze glycosidic bonds, and peptidase A1 (IPR001461) were highly enriched (*P*<0.05) in the species-specific secretomes of *Py. aphanidermatum* (Table S10). In addition, cutinase (IPR000675), glycoside hydrolases (IPR002594, IPR001137), and peptidase inhibitors (IPR011497, IPR013201) domains were enriched in *Py. arrhenomanes-*specific secretomes relative to their proteomes. The enrichment and their expression upon infection in plant pathogenic oomycetes have already been shown for different families of hydrolases and lyases [55], [60]. The NPP1 domain (IPR008701) that is present in necrosis-inducing proteins was highly enriched in the *Py. aphanidermatum* and *Py. irregulare*-specific secretomes. The necrosis-inducing proteins are known for their ability to trigger numerous plant defense responses, necrosis, and cell death in dicotyledonous plants [70]. Several transporter-related domains (IPR005828, IPR003439, IPR011547) along with peptidase S8/S53 (IPR000209) were highly enriched in *Py. vexans*-specific secretome. The membrane transporter (e.g. ABC transporter) may play important role in counteracting the physiological impact of host defense compounds [71]. Domains containing leucine-rich repeat (IPR001611) were highly enriched in *Py. iwayamai* along with several peptidase domains (IPR001506, IPR001394, IPR001461). The *Py. irregulare*-specific secretome was highly enriched for peptidase A1 (IPR001461), pectinesterase (IPR000070), NPP1 domain (IPR008701), serine protease inhibitor (kazal-type (IPR011497)). The protein domains specifically enriched in different *Pythium* species (e.g., certain transporter families, peptidase, and domains related to metabolism of carbohydrates) highlight the differences between these groups of plant pathogens in terms of their pathogenicity and host preference.

### RxLR Effectors

The genomes of the three *Phytophthora* species encode large numbers (350 to 563) of potential effector proteins that are implicated in pathogenesis [9], [72], [73]. These proteins contain a conserved amino-terminal cell entry domain with the motifs RxLR and dEER [6], [7], which mediate their entry into host cells [74], [75], [76]. RxLR-dEER effectors are hypothesized, and in a few cases experimentally shown, to suppress host defense responses [77]. However, some of these effectors can be recognized by plant immune receptors resulting in programmed cell death and disease resistance [78], [79]. Although no RxLR effectors are present in the *Py. ultimum* var. *ultimum* genome [53], evolution under diverse environmental conditions and co-evolution with diverse hosts could lead to inter-specific variation in RxLR effectors among *Pythium* species.

We used four different bioinformatics approaches to ascertain if RxLR effector genes occurred within the genomes of the six *Pythium* species sequenced in this study. Consistent with previous analyses of the *Py. ultimum* var. *ultimum* genome [53] in which no RxLR effectors were detected, we failed to identify any candidate effectors in any of the six *Pythium* species sequenced. Our results suggest that in all seven of the *Pythium* species surveyed, RxLR effectors are absent signifying substantial differences in virulence and the interaction of *Pythium* species with plant hosts as compared to *Phytophthora* and *Hyaloperonospora* species (Table S7). Since *Phytophthora* genomes have 350–563 RxLR effector candidates [6], [7], the absence of these effectors in *Pythium* genomes indicates that the effectors are not required for virulence of *Pythium* species. As compared to hemibiotrophic *Phytophthora* species, *Pythium* species are adapted to necrotrophic lifestyle and may not require RxLR effectors for successful colonization and establishment of the infection structure. Other effectors such as secreted hydrolytic enzymes and necrotizing toxins may play important role during necrotrophy.

### YxSL[RK] Candidate Effectors

**Figure 4:**
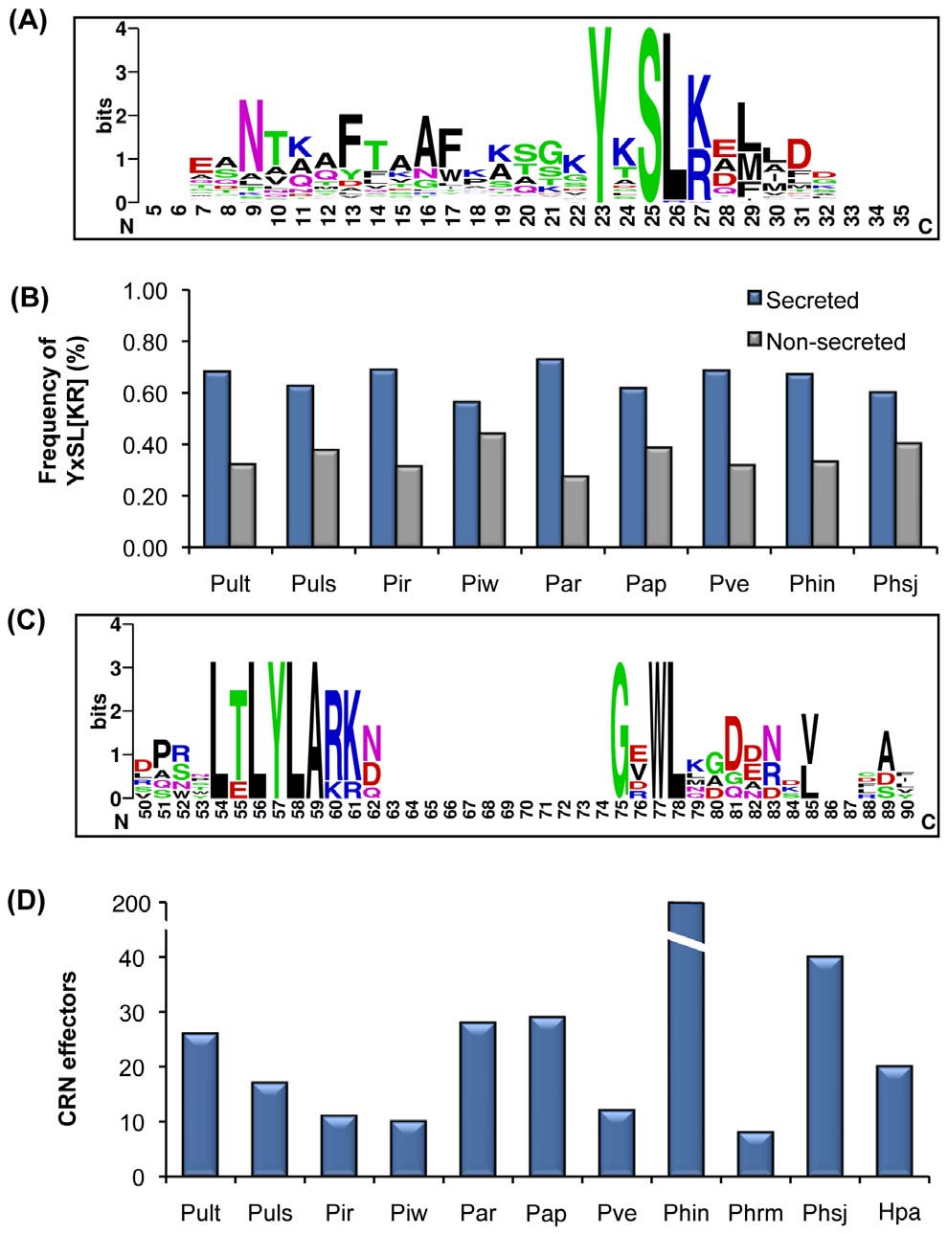
Candidate effector proteins from *Pythium*. (A) The typical architecture of a YxSL[RK] effector candidate inferred from 141 sequences from seven *Pythium* species, *Phytophthora infestans*, and *Phytophthora sojae*. The consensus sequence pattern of the YxSL[RK] motif was calculated using WebLogo [103]. The bigger the letter, the more conserved the amino acid site. Please note that the numbers in the sequence logo refer to the corresponding positions in the alignment and thus differ from the average position of the motifs. (B) The YxSL[RK] motif distribution in the proteomes of *Pythium* species, *Phytophthora infestans* and *Phytophthora sojae* is shown. The YxSL[RK] sequence is over-represented in the secretome of *Pythium* and *Phytophthora* species relative to the non-secreted proteome (*P*≤0.05). The YxSL[RK] motifs were counted only if they were within the first 30 to 150 residues from the signal peptide. The frequency was calculated as percentage of either all secreted proteins or all non-secreted proteins. Pult, *Pythium ultimum* var. *ultimum*; Puls, *Pythium ultimum* var. *sporangiiferum*; Pir, *Pythium irregulare*; Piw, *Pythium iwayamai*; Par, *Pythium arrhenomanes*; Pap, *Pythium aphanidermatum*; Pve, *Pythium vexans*; Phin, *Phytophthora infestans*; Phrm, *Phytophthora ramorum*; Phsj, *Phytophthora sojae*; Hpa, *Hyaloperonospora arabidopsidis*. (C) The typical architecture of an LxLYLAR/K effector motif inferred from 129 sequences from 7 *Pythium* species. The consensus sequence pattern of the LxLYLAR/K motif was calculated using WebLogo [103]. The bigger the letter, the more conserved the amino acid site. Please note that the numbers in the sequence logo are referring to the corresponding positions in the alignment and thus differ from the average position of the motifs. (D) Number of CRN effector proteins in oomycetes. The number of candidate CRN effectors estimated by Hidden Markov Model (HMM) searches in combination with two other computational methods is shown. The number of CRN effectors from *Pythium ultimum* var. *ultimum*, *Phytophthora* species and *H. arabidopsidis* were taken from published genome datasets [6], [7], [52], [53]. Pult, *Pythium ultimum* var. *ultimum*; Puls, *Pythium ultimum* var. *sporangiiferum*; Pir, *Pythium irregulare*; Piw, *Pythium iwayamai*; Par, *Pythium arrhenomanes*; Pap, *Pythium aphanidermatum*; Pve, *Pythium vexans*; Phin, *Phytophthora infestans*; Phrm, *Phytophthora ramorum*; Phsj, *Phytophthora sojae*; Hpa, *Hyaloperonospora arabidopsidis*.

The YxSL[RK] class of putative effectors have been found in many pathogenic oomycetes including *Py. ultimum* var. *ultimum* [53], [80], [81]. Interestingly, the YxSL[RK] motif shares similarity in sequence and position with the canonical RxLR motif and appears to be a signature for a novel family of secreted proteins that may function as effectors [81] in *Pythium* and *Phytophthora* species. We computationally screened our newly sequenced six *Pythium* genomes for candidate YxSL[RK] effectors using a HMM profile of a putative YxSL[RK] motif constructed using 57 genes containing the corresponding motif from *Py. ultimum* var. *ultimum* [53], three *Phytophthora* species, and *Aphanomyces eutieches* [82]. Proteins with the YxSL[RK] motif situated within 30 to 150 amino acids positions after the initial methionine were considered for further analyses. Using the YxSL[RK] motifs previously identified in *Py. ultimum* var. *ultimum* as a positive control [53], we identified an initial set of 123 proteins containing the YxSL[RK] motif in the six *Pythium* species. After searching against the HMM profile and multiple sequence alignment of the 123 proteins, we removed three proteins with an YxSL[RK] motif positioned outside the 30 to 150 amino acid position range. Using the same HMM profile, we were able to identify 21 additional proteins containing the YxSL[RK] motif from *Ph. infestans* and *Ph. sojae*. Alignment of the core set of 141 YxSL[RK] effectors from *Pythium* and *Phytophthora* show a modular organization with a conserved amino-terminal region, containing four conserved motifs, followed by a highly variable carboxy-terminal region as reported for other oomycete effectors [9] (Figure 4A). The YxSL[RK] candidates are significantly enriched (*P*≤0.05) within the secretome of *Ph. infestans*, *Ph. sojae,* and all seven *Pythium* species (Figure 4B), nearly two-fold higher in the secreted proteome as compared to the rest of the proteome.

### CRN Effectors

The genomes of many oomycete pathogens harbor a large repertoire of a class of candidate effectors termed “Crinklers” (CRN) that are presumed to enter host cytoplasm [6], [83], [84] and elicit necrosis *in planta* [83]. First identified in *Phytophthora* based on their ability to elicit plant cell death and defense responses [83], these effectors have been identified in all phytopathogenic oomycete genomes sequenced to date [6], [7], [52], [53], [85]. Similar to the RxLR effectors, CRN effectors contain a conserved motif, LFLAK, following the signal peptide [6], [84]. Contrary to RxLR effectors, CRN effectors are present in all oomycete plant pathogen genomes suggesting that these are an evolutionarily conserved set of effectors in phytopathogenic oomycetes, including *Py. ultimum* var. *ultimum* [53]. Through BLASTP [86] searches using 21 well-defined amino-terminal domains from *Ph. infestans* and *Py. ultimum* var. *ultimum,* we identified 45 predicted CRN proteins in the six newly sequenced *Pythium* species. We aligned all predicted *Pythium* CRN sequences with the CRN proteins from *Ph. infestans* and *Py. ultimum* var. *ultimum* to build an HMM profile and using HMMer we identified 53 additional candidate effectors with an LFLAK-like domain in other *Pythium* species. Using the same HMM profile built from alignment of *Pythium* and *Phytophthora* CRN sequences, we were able to identify 14 of the 20 candidate CRN effector proteins from the *H. arabidopsidis* genome [52]. Further string searches of the *Pythium* proteomes using LxLFLAK and LxLYLAR/K, a conserved motif that is shared amongst *Py. ultimum* var. *ultimum* CRN proteins [53], resulted in identification of 5 additional predicted proteins with LxLFLAK-like domains from the six *Pythium* species. Examination of a set of 129 predicted effector proteins (including 26 from *Py. ultimum* var. *ultimum*) from all *Pythium* species revealed a conserved LxLYLAR/K motif followed by conserved WL motif (Figure 4C) that is shared amongst CRN proteins. Consistent with previous results, the LxLYLAR/K motif was located between 40 and 65 amino acids after the initial methionine, followed by an adjacent diversified WL domain reflecting the modular design of CRN proteins in the oomycetes [6]. Given the important functions of effectors in oomycete pathogenicity, we compared the number of CRN effector classes across oomycete species for which genome sequences are available. Surveys of genome sequences showed that every examined species, including *Albugo laibachii*, and *H. arabidopsidis*, have candidate CRN genes [6], [7], [52], [53], [85] indicating that these effectors are ubiquitous in plant pathogenic oomycetes [6], [7], [52], [53], [85]. Comparison of the number of these effectors shows expansion in *Ph. infestans* [6] and high intraspecific variation in *Pythium* similar to those found in *Phytophthora* species (Figure 4D). The intraspecific variation in number of CRN effector indicates that the *Pythium* species may have adopted species-specific strategies for infection and these strategies could be important during their interaction with different hosts.

### Comparative Genomics

#### Shared gene clusters of oomycetes

The *Straminipila* includes phytopathogenic oomycetes and autotrophic diatoms. A phylogenetic tree constructed using the Bayesian analyses of nuclear large subunit of rDNA from 14 stramenopiles shows five broad clades: the *Phytophthora* species with *Hyaloperonospora* and *Py. vexans*, the *Pythium* species with globose sporangia, the *Pythium* species with filamentous sporangia, the diatom group with *T. pseudonana* and *P. tricornutum*, and a separate clade of *S. parasitica* (Figure 1).

**Figure 5:**
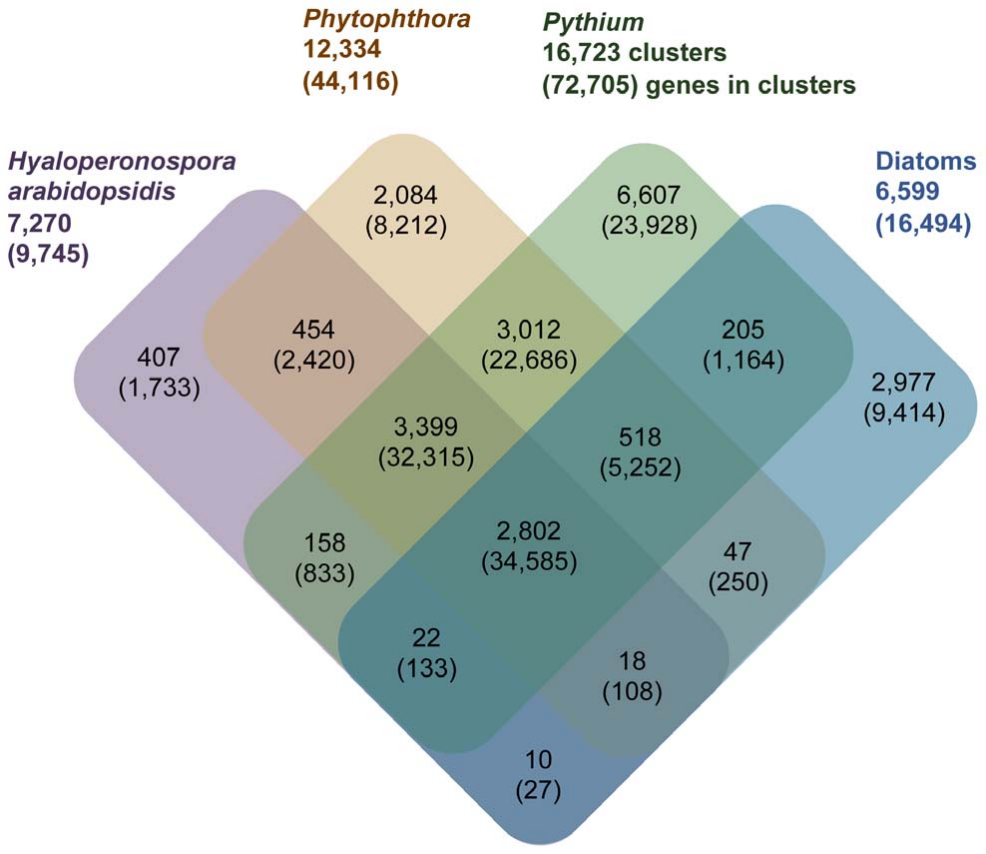
Orthologous gene families of *Pythium* species, *Phytophthora* species, *Hyaloperonospora arabidopsidis* and two diatom species. Protein-coding genes from seven *Pythium*, three *Phytophthora*, two diatom species and *Hyaloperonospora arabidopsidis* were clustered using OrthoMCL [59]. The number of gene families (clusters) and the total number of clustered genes (in parentheses) are indicated for each taxon and their interactions in the Venn diagram. The numbers outside the Venn diagram indicate the total number of orthologous clusters and number of genes (in parentheses) in the clusters for each taxon.

To identify common features in all oomycete genomes, we compared the gene family content of the 11 phytopathogenic oomycetes and two autotrophic diatoms using OrthoMCL [59] using the predicted protein-coding genes from these 13 species. A total of 182,894 protein-coding genes from *Pythium* (7 species), *Phytophthora* (3 species), *H. arabidopsidis,* and diatoms (2 species) were clustered revealing the stramenopile-core (clusters shared by all 13 taxa), *Pythium*/*Phytophthora*-specific (including clusters specific to *Phytophthora* and *Pythium*), Hpa-specific (clusters specific to *H. arabidopsidis*) and diatom-specific clusters (clusters specific to two diatom species, Figure 5). A total of 143,060 genes were clustered into 22,720 gene families (clusters) while 39,834 genes were singletons. The core stramenopile cluster of 2,802 gene families contained 34,585 genes. The stramenopile-core genes showed over-representation of major facilitator superfamily (IPR011701) and amino acid transporter, transmembrane (IPR013057) domains, with significant under-representation of glycoside hydrolases (IPR000743, IPR001139), protease inhibitors (Kazal-type (IPR011497)), and necrosis inducing (IPR008701) domains (Bonferroni-corrected *P*<0.05) relative to the non-core genes (genes other than core genes) from stramenopiles (Table S11). Comparatively higher numbers of diatom-specific genes (2,977 clusters, 9,414 genes) is consistent with the specialized adaptation of diatoms to a phototrophic lifestyle compared to phytopathogenic oomycetes. Kinase-encoding domains (IPR000719, IPR001245) were highly represented in diatom-specific genes while domains including HECT ubiquitin ligase (IPR000569), peptidase M16, C-terminal (IPR007863), proteinase inhibitor I29 (cathespin propeptide (IPR013201)), and glycoside hydrolase, family 30 (IPR001139) were depleted. The *Pythium*-*Phytophthora*-specific gene sets (3,012 clusters, 22,686 genes) were highly enriched with protein domains possibly related to pathogenesis including necrosis inducing (IPR008701), elicitin (IPR002200), glycoside hydrolases (IPR000743, IPR011583), and peptidases (IPR001577, IPR001563) (*P*<0.05) as compared to the rest of the genes from *Pythium* and *Phytophthora*. For several groups of over-represented domains in the *Pythium*-*Phytophthora*-specific genes, a direct or indirect role in host-pathogen interaction and/or plant pathogen lifestyle has already been hypothesized or demonstrated [6], [55], [60], [87]. Several secreted protease inhibitors (Kazal-type (IPR011497)), peptidase M8 (leishmanolysin (IPR001577)), and peptidase S10 (serine carboxypeptidase (IPR001563)) domains are significantly over-represented in *Pythium*-*Phytophthora*-specific clusters, suggestive of a role in protection of the pathogen against host-encoded defense-related proteases as shown in other oomycete pathogens [55]. In comparison to the rest of the genes from *H. arabidopsidis*, the *H. arabidopsidis*-specific genes (407 clusters, 1,733 genes) showed under-representation of domains related to transport (IPR011547), host-targeted degradative enzyme (IPR021067), elicitin (IPR002200), and necrosis-inducing protein (IPR008701) (P<0.05) (Table S11). It is possible that in evolving a biotrophic lifestyle, *H. arabidopsidis* lost many secreted hydrolytic enzymes [52].

### Syntenic Relationships among Oomycetes

The availability of several *Pythium* genome sequences permits the first detailed investigation of genome evolution within the genus and comparison with that of other straminopiles. By comparison with *Py. ultimum* var. *ultimum*, we identified syntenic regions across stramenopiles and analyzed rearrangements in gene order. Previous analyses of synteny between selected regions of *Py. ultimum* var. *ultimum* and *Phytophthora* species showed very similar ortholog content in broad regions but a high level of rearrangement in local gene order [53]. Here, we expanded the syntenic analyses to six other *Pythium* species, *H. arabidopsidis* and the diatom *T. pseudonana*. Due to the fragmented nature of the assemblies in all of the genomes, we identified contigs or scaffolds with a minimum of five genes and identified syntenic blocks in comparison to the *Py. ultimum* var. *ultimum* genome using MCscan [88]. A contig or scaffold was considered syntenic if at least three genes in a five-gene block was co-linear with *Py. ultimum* var. *ultimum* genome. The comparison of *Pythium* genomes shows a varying degree of synteny between *Py. ultimum* var. *ultimum* and six other *Pythium* species. Among all *Pythium* species, *Py. ultimum* var. *ultimum* was most syntenic with *Py. ultimum* var. *sporangiiferum* and least syntenic with *Py. arrhenomanes* and *Py. iwayamai* followed by *Py. aphanidermatum* (Table S12).

**Figure 6:**
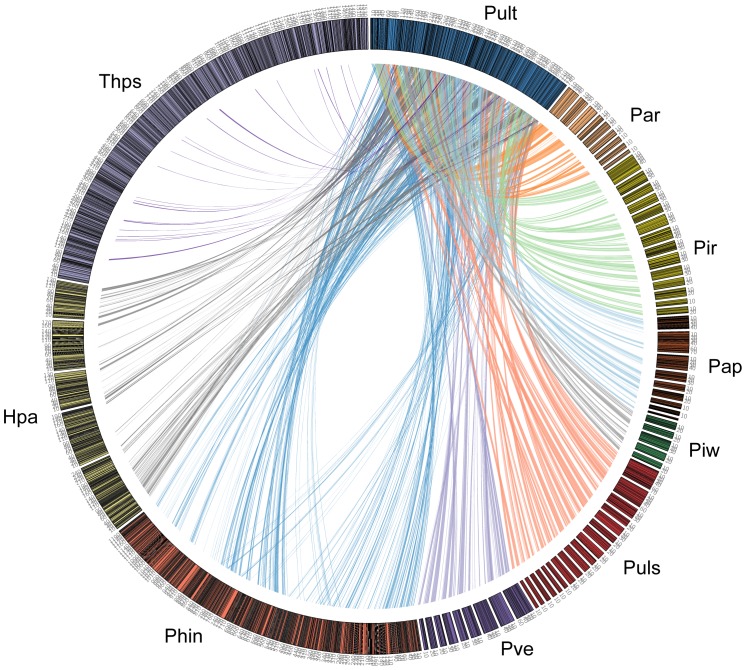
Syntenic analyses of stramenopile genomes. The circle is a graphical representation of the selected regions from *Pythium arrhenomanes* (contigs 8, 17, 26, 41, 68, 131, 170, 285, 707) *Pythium irregulare* (contigs 28, 92, 103, 106, 119, 123, 129, 132, 140, 163, 195, 226, 372, 396), *Pythium aphanidermatum* (scaffolds 4, 6, 23, 80, 88, 96, 115, 150, 327), *Pythium iwayamai* (contigs 18, 28, 29, 61, 235), *Pythium ultimum* var. *sporangiiferum* (contigs 4, 6, 34, 106, 121, 134, 150, 173, 181, 222, 231, 257, 319, 404, 437, 458, 533, 726), *Pythium vexans* (contigs 9, 31,42, 94, 151, 160, 209, 220, 347), *Phytophthora infestans* (supercontig 1.2), *Hyaloperonospora arabidopsidis* (scaffolds 5, 6, 7, 8, 9) and *Thalassiosira pseudonana* (chromosome 3). Numbers along each ideogram are sequence lengths in kbp. Syntenic regions were identified through reciprocal best matches between gene models and block identification using MCscan [88]. Each line radiating from *Py. ultimum* var. *ultimum* (scf1117875581354) links a syntenic gene pair. Each species is represented by a genus-species abbreviation and colored as *Pythium ultimum* var. *ultimum* (Pult) in blue, *Pythium arrhenomanes* (Par) in orange, *Pythium irregulare* (Pir) in yellow, *Pythium aphanidermatum* (Pap) in dark brown, *Pythium iwayamai* (Piw) in green, *Pythium ultimum* var. *sporangiiferum* (Puls) in dark red, *Pythium vexans* (Pve) in purple, *Phytophthora infestans* (Phin) in brick red, *Hyaloperonospora arabidopsidis* (Hpa) in olive green, and *Thalassiosira pseudonana* (Thaps) in light purple.

In order to examine the conservation of gene order across three oomycete genera, we compared *Py. ultimum* var. *ultimum* not only with the other six *Pythium* species but also with *Ph. infestans, H. arabidopsidis,* and *T. pseudonana*. Figure 6 shows the conservation of gene order between one of the largest scaffolds from *Py. ultimum* var. *ultimum* (scf1117875581354) with *Ph. infestans* (supercontig 1.2), *H. arabidopsidis* (scaffolds 5,6,7,8 and 9) and *T. pseudonana* (chromosome 3). As expected, the gene order was highly conserved among *Pythium* species. The level of synteny revealed by our analyses extends the previously reported synteny between *Py. ultimum* var. *ultimum* and *Phytophthora* species [53] unveiling conservation of a portion of gene order not only within seven *Pythium* species but also between other stramenopiles. Within oomycetes, the conservation of synteny between species recapitulates the phylogeny shown in Figure 1. The overall degree of conservation is high, being highest among the most closely related species (as shown by the larger spans with fewer breaks in synteny between *Pythium* species) than with distantly related species (*e.g. H. arabidopsidis* and *T. pseudonana*).

## Conclusions

The genome sequences of 13 stramenopiles enabled genome-wide comparison of gene repertoires within and between phytopathogenic oomycetes and non-pathogenic diatoms. Our comparative analyses of stramenopiles indicate that developmental innovations in oomycete pathogens involve secretion of a large number of effector molecules, proteolytic enzymes, and cell wall hydrolyzing enzymes. However, expansion of a suite of genes encoding effectors and proteolytic enzymes reflect specific adaptations to trophic lifestyle.

These comparative analyses revealed some of the genetic mechanisms underlying necrotrophic and biotrophic lifestyle. The hemibiotrophic *Phytophthora* species show expansion and diversification of protein families associated with plant infection such as some glycoside hydrolases, ABC transporters and in particular, oomycete pathogenesis related genes. In contrast to the biotrophic *H. arabidopsidis*, which exhibits dramatic reductions in genes encoding RxLR effectors and other secreted pathogenicity proteins, cell wall hydrolytic enzymes and transporters, the non-biotrophic group (*Phytophthora* and *Pythium*) seems to have a large suite of pathogenicity related genes, as a result of expansion of effector families in *Phytophthora* and proteolytic enzymes in *Pythium* (Table S7). These differences in rich repertoires of candidate effectors could underlie the coevolution and adaptation of these pathogens to the plant immune system and set them apart from the non-pathogenic autotrophic stramenopiles (e.g. diatoms). A deeper understanding of the complex array of factors, including secreted proteins and proteolytic enzymes identified in this study, which affect host-pathogen interactions and coevolution, could enable efficient targeting of pathogen-control measures in agricultural ecosystems.

## Materials and Methods

### Illumina Sequencing and Assembly

Approximately 1 g of freshly harvested mycelium from *Pythium* species was ground in a FastPrep® (BIO 101) machine (Savant, Inc. Holbrook, NY) and the genomic DNA was extracted following a protocol modified from Moller *et al.* [89]. Libraries were constructed from genomic DNA of *Py. arrhenomanes* (ATCC 12531 = CBS 324.62), *Py. irregulare* (DAOM BR486 = CBS 250.28), *Py. iwayamai* (DAOM 242034 = CBS 132417), *Py. ultimum* var. *sporangiiferum* (DAOM BR650 = CBS 219.65), and *Py. vexans* (DAOM BR484 = CBS 119.80) using the Illumina Genomic DNA Sample kit (Illumina, San Diego, CA). The libraries were paired-end sequenced (76 or 120 bp) on the Illumina Genome Analyzer II/IIx sequencer. The purity-filtered (PF) reads were first trimmed and then quality filtered using custom Perl scripts to remove reads with low quality bases (<Q20) (Table S1).

The trimmed and quality filtered reads were then assembled using Velvet v0.7.63 [90] in conjunction with the VelvetOptimiser tool v2.10 [91], a multi-threaded Perl script for automatically optimizing the parameter options for the Velvet assembler. VelvetOpimiser was run with a k-mer range of 21 to 41 for each assembly the final assembly parameters for each assembly is in Table S2.

### Pyrosequencing and Assembly

For *Py. aphanidermatum* (DAOM BR444 = CBS 132490), a single-end and a paired-end (3 Kb) library was created and sequenced using the Genome Sequencer FLX instrument following the manufacturer’s protocol (Roche Applied Science, Mannheim, Germany). The paired-end library was sequenced using the Titanium chemistry. The single-end library yielded 1,299,108 reads (507 Mb) with an average length of 390 bp. The paired-end library yielded 380,566 reads (137.5 Mb) with an average length of 361 bp. 256,098 of these reads were indentified as a member of a valid read pair. The reads were assembled with the Newbler assembler v2.3 [92] with the large genome mode and paired-end mode flags set. The final assembly statistics is summarized in Table 2 and Table S3.

### Genome Annotation

The assembled genomes were annotated using the MAKER v2.03 [93] annotation pipeline. A *Pythium* specific repeat library constructed previously [53] was supplied to MAKER for the repeat masking step. Gene calls were generated using FGENESH [94] using the *Phytophthora* matrix and SNAP [95], which was trained using the transcripts from the *Pythium ultimum* Genome Database (http://pythium.plantbiology.msu.edu/). All the oomycete ESTs in dbEST [96] and all the oomycete proteins in GenBank were provided to MAKER as evidence to refine the annotation. The final annotation set produced by MAKER is summarized in Table 2 and Table S3.

### Phylogenetic Analysis

The phylogeny of 14 stramenopiles was created by using 123 ITS rDNA sequences obtained from GenBank. Nucleotide sequences were aligned by ClustalW [97]. Phylogenetic analyses were performed using the MrBayes program for Bayesian analysis [98] using Markov Chain Monte Carlo (MCMC) with the general time reversible (GTR) model. The program was run for 1,000,000 generations and sampled every 100 generations. Phylogenetic tree was visualized in Mega5 [99].

### Identification of Orthologous Groups

Orthologous and close paralogous genes were identified using OrthoMCL v1.4 [59] with default parameters. Protein domains were predicted by InterProScan [100]. For each genome or group specific proteins, the total number of proteins with each type of domain was computed.

### Identification of Putative Secreted Proteins

Signal peptides were predicted using SignalP v3.0 [64] and transmembrane domains predicted with TMHMM [65]. Proteins showing (i) SignalP3.0 NN Ymax Score ≥0.5 and (ii) SignalP3.0 NN D-score ≥0.5 and (iii) SignalP3.0 HMM S probability ≥0.9 and (iv) predicted localization “Secreted” (S) and (v) no TMHMM predicted transmembrane domain after signal peptide cleavage site were considered to be within the *Pythium* secretome. Sequences that were predicted to contain transmembrane domains or organelle-targeting signals were omitted from the secretome. The clustering of secreted protein was done using OrthoMCL v1.4 [59].

### Enrichment Analyses

InterProScan [100] with default parameters were used to complement the annotation of the secreted proteins. GO terms were assigned using Blast2GO [68] with default parameters. Enrichment frequencies in the core *Pythium*, stramenopile-core and taxa-specific gene families were calculated as the number of occurrences over the total number of protein domain or GO hits among secreted versus non-secreted proteins. Significance of enrichment/depletion is assessed by a Chi-square test with Bonferroni correction for multiple testing. Only protein domains with enrichment p-value≤0.05 and GO with enrichment p-value≤0.05 were considered.

### Carbohydrate-active Enzyme Analyses

The carbohydrate-active enzyme coding genes of *Pythium*, *Phytophthora, H. arabidopsidis* and diatom genomes were automatically annotated using the CAZymes Analysis Toolkit – CAT [62] according to the CAZy database classification [61]. Enzyme annotation was done using two approaches. First, a bi-directional BLAST search was performed against the entire non-redundant sequences of the CAZy database. Second, a link or correspondence between the CAZy families and protein family domains was analyzed. A manual scan was also performed based on the PFAM domain information.

### Identification of Candidate Effectors

The candidate RxLR effectors were identified using the approach described by Win *et al.* [101]. We used four different bioinformatics approaches to identify the predicted set of effectors. First, we translated all six frames of the *Pythium* genome sequences to identify proteins with an amino-terminal signal peptide based on SignalP prediction using SignalP v3.0 [64] with a SignalP HMM score cutoff of ≥0.9. The transmembrane domains were predicted with TMHMM [65] and sequences that were predicted to contain transmembrane domains or organelle-targeting signals were omitted. Candidate RxLR effectors were selected from these secreted translations using custom Perl scripts. Secreted translations with RxLR position between 30 and 150 residues from signal peptide, RxLR position downstream of the signal peptide cleavage site and SignalP v3.0 NN predicted cleavage site of less than 30 amino acids were selected as candidate RxLR effectors. The six frame translation of *H. arabidopsidis* genome which is reported to have 134 candidate RxLR effectors was used as a positive control. Second, an HMM profile of the RxLR domain was constructed by manually aligning the RxLR domains of the 53 RxLR effectors from *Phytophthora* species and *H. parasitica*. The resulting alignment was fed to the hmmbuild program (HMMer software) [102] to generate the HMM profile. The HMM profile was used to search the translations for candidate effectors using the hmmsearch program [102]. To validate our computational approach, the same HMM profile was used to search the six frame translation of *H. arabidopsidis* genome. Furthermore, the whole *Pythium* proteome was also searched with the HMM. Third, a comprehensive database of RxLR effector proteins from *Phytophthora* species [6], [7], *H. arabidopsidis* [52], and *A. laibachii* [85] was created. Putative homologs in predicted proteomes of *Pythium* were identified by BLASTP search against the RxLR effector database at E-value cutoff of 10^−5^. Fourth, a string searches was performed for the RxLR, RxLx and Rx[LMFY][HKR] motif within the amino terminus of each six frame translation of *Pythium* genomes, 30 to 150 residues from the signal peptide.

We computationally screened the six *Pythium* genomes for candidate YxSL[RK] effectors using a HMM profile of the putative YxSL[RK] motif, a novel effector motif identified first in *Py. ultimum* var. *ultimum*, constructed using 57 genes containing YxSL[RK] motif from three *Phytophthora species* and *Aphanomyces eutieches*. Using the YxSL[RK] motifs from *Py. ultimum* var. *ultimum* as a control, we identified an initial set of 123 proteins containing the YxSL[RK] motif in 7 *Pythium* species. Using the same HMM profile we were able to identify 21 additional proteins with the YxSL[RK] motif in the *Ph. infestans* and *Ph. sojae* genomes. After searching against the HMM profile and multiple alignment of the proteins we selected a set of 141 proteins, which includes 120 candidate effectors from seven *Pythium* species and 21 from two *Phytophthora* species, with YxSL[RK] motif situated between 30 to 150 amino acids positions.

For the CRN effectors, a BLASTP search against 21 well-defined amino-terminal domains from *Ph. infestans* and *Py. ultimum* var. *ultimum* CRN sequences was performed to identify proteins with putative LFLAK-like domains. The candidate CRN sequences from *Ph. infestans* and *Pythium* species were used to construct an HMM profile and the CRN sequences from *Py. ultimum* var. *ultimum* were used as a control. Two criteria were used to identify candidate LxLYLAR/K proteins. First, the conserved motif should be preceded by a signal peptide and followed by WL motif. Second, the conserved motif should be located between 40 to 65 amino acids after first methionine. Using the HMM profile, we identified additional candidate effectors with an LxLYLAR/K domain. To validate our computational methods, the same HMM profile was used to identify the CRN effectors from *H. arabidopsidis* genome which is reported to have 20 candidate CRN effectors [52]. Multiple alignments were conducted using the programs ClustalW and ClustalX [97]. Sequence alignments were submitted to the WebLogo server [103] to generate a sequence logo for the consensus.

### Transcriptome Sequencing

One pooled cDNA library was constructed for each of four *Pythium* species (*Py. arrhenomanes*, *Py. irregulare*, *Py. iwayamai,* and *Py. vexans*). Initially, plugs of 10% V8 agar containing *Pythium* species were incubated for 1 day in yeast extract broth (YEB; 30 g/l sucrose, 1 g/l KH_2_PO_4_, 0.5 g/l MgSO_4_·7 H_2_O, 0.5 g/l KCl, 10 mg/l FeSO_4_·7 H_2_O, 1 g/l yeast extract) at 25°C with shaking (200 rpm). Approximately 50 mg of hyphae growing out of the plugs were then transferred to flasks containing media for the various expression assays, with the exception of *Py. iwayamai,* mycelium was grown under the following conditions: 1, nutrient-rich YEB medium for 3 days at 25°C with shaking (200 rpm); 2, and nutrient-starved Plich medium (S Kamoun, unpublished) for 10 days at 25°C in standing culture, as previously described [104]; 3, YEB medium for 2 days at 25°C with shaking (200 rpm) followed by the addition of 1 and 100 µl/l of the fungicide mefenoxam (Subdue MAXX™, Novartis Crop Production, Greensboro, NC, USA) and subsequent incubation for an additional 3 hours at the same temperature and with agitation; 4, YEB medium for 2 days at 25°C with shaking (200 rpm) followed by a cold stress of 0°C with shaking (200 rpm) for 6 hours; 5, YEB medium for 2 days at 25°C with shaking (200 rpm) followed by a heat stress of 35°C for 6 hours. *Py. iwayamai* was isolated from a cool temperature site and therefore incubated at 10°C and was not exposed to above mentioned conditions. Condition 5 was not included, because *Py. iwayamai* did not resist the heat stress at 35°C. For each condition listed above, mycelium was harvested, macerated in liquid nitrogen and RNA was extracted as described [104]. RNA was treated with DNAse (Promega RQ1 RNase-Free DNase, Madison, WI, USA) and subsequently pooled in an equimolar manner with the RNA from other conditions. From each pooled library 10 µg RNA was used to construct cDNA using the mRNA-Seq Sample Prep Kit (Illumina, San Diego, CA, USA), which was sequenced with Illumina Genome Analyzer (GA) II using version 3 sequencing reagents for 36 cycles. Base calling was carried out using the Illumina GA pipeline v1.4.

For each library, the purity filtered reads from the Illumina Genome Analyzer II pipeline were mapped using Tophat v1.1.4 [105], which works in conjunction with Bowtie, a short read aligner v0.12.7 [106]. The minimum and maximum intron sizes were 5 bp and 15 kbp, respectively, for each Tophat run. The final annotation GFF3 file was provided to Tophat and expression values (FPKM) were calculated using the Cufflinks package v0.9.3 [107]. A gene was considered expressed if the FPKM value and FPKM 95% confidence interval lower boundary was greater than 0.001 and zero, respectively.

### Synteny Analyses

All protein coding genes from the seven *Pythium* genomes, *Ph. infestans*, *H. arabidopsidis,* and *T. pseudonana* were compared to each other via an all-by-all BLASTP [108] to generate the appropriate input for the MCscan algorithm [88] (BLASTP, E-value≤1e-10). A python script contained in the MCscan package was used to filter the BLASTP output to remove self-matches and to reorder the list of gene pairs. MCscan v0.8 was used to calculate synteny between all combinations of genomes using the pooled BLASTP output and the genomic coordinates. A minimum of 3 genes within a 5 gene block was required to constitute a syntenic block (default MCscan value is 5). The size of the search window was calculated by MCscan based on the average intergenic distance in the genomes being compared. Default values were used for all other parameters. Each syntenic block is assigned an E-value by MCscan. Custom PERL scripts were used to parse the MCscan output and calculate the total number of syntenic blocks for each genome combination. MCscan output was parsed to create files appropriately formatted for input to Circos [109] for visualization. Figure 6 shows an example spanning selected syntenic regions of *Pythium*, *Ph. infestans*, *H. arabidopsidis,* and *T. pseudonana* genomes. Each syntenic block is represented as a link whose ends represent the syntenic regions from other species.

### Data Access

The whole genome shotgun projects have been deposited at DDBJ/EMBL/GenBank under the accession numbers: AKXX00000000 for *Py. aphanidermatum*, AKXY00000000 for *Py. arrhenomanes*, AKXZ00000000 for *Py. irregulare*, AKYA00000000 for *Py. iwayamai*, AKYB00000000 for *Py. ultimum* var. *sporangiiferum*, and AKYC00000000 for *Py. vexans*. The genome assemblies, transcript sequences, and protein sequences are also available for download and BLAST searching at Pythium Genome Database (PGD) website (http://pythium.plantbiology.msu.edu/, see download and BLAST pages). Also available for download at the PGD are the annotation files in GFF3 format and the functional annotation of the gene models (http://pythium.plantbiology.msu.edu/download.shtml). The genome assembly and annotation files are also available for download from the Dryad Digital Repository (http://dx.doi.org/10.5061/dryad.h748p). The WGS reads are available in the NCBI Short Read Archive (SRA) under the accession SRP006957. The RNA-Seq Reads are available the NCBI SRA under the accession SRP006964.

## Supporting Information

Figure S1 **Shared clusters of secreted proteins in** ***Pythium*** **.** The Venn diagram shows the distribution of secreted protein clusters among *Pythium* species. The putative secreted proteins from seven *Pythium* species were predicted by using SignalP v3.0 [64] and clustered using OrthoMCL [59]. The number of gene families (clusters) and the total number of clustered genes (numbers in parentheses) are indicated.
(TIF)

Table S1 **Summary of sequence reads generated for five** ***Pythium*** **species.** Sequencing was done using the Illumina Genome Analyzer (GA) II or IIx platform. PF, purity-filtered; Par, *Pythium arrhenomanes*; Pir, *Pythium irregulare*; Piw, *Pythium iwayamai*; Puls, *Pythium ultimum* var. *sporangiiferum*; Pve, *Pythium vexans*.
(XLS)

Table S2 **Assembly parameters for five** ***Pythium*** **species.** Sequenced reads were assembled using Velvet v0.7.63 [90] in conjunction with the VelvetOptimiser tool v2.10 [91]. Par, *Pythium arrhenomanes*; Pir, *Pythium irregulare*; Piw, *Pythium iwayamai*; Puls, *Pythium ultimum* var. *sporangiiferum*; Pve, *Pythium vexans.*
(XLS)

Table S3 **Assembly and annotation statistics of thirteen stramenopile genomes.** Assembly and annotation statistics were compared to data published by Levesque *et al*. for *Pythium ultimum* var. *ultimum* [53], Haas *et al*. for *Phytophthora infestans* [6], Tyler *et al.* for *Ph*ytophthora *ramorum* and *Ph*ytophthora *sojae* [7], Baxter *et al*. for *Hyaloperonospora arabidopsidis* [52], Armbrust *et al*. for *Thalassiosira pseudonana* [58] and Bowler *et al*. for *Phaeodactylum tricornutum* [57]. Pap, *Pythium aphanidermatum*; Par, *Pythium arrhenomanes*; Pir, *Pythium irregulare*; Piw, *Pythium iwayamai*; Puls, *Pythium ultimum* var. *sporangiiferum*; Pult, *Pythium ultimum* var. *ultimum*; Pve, *Pythium vexans*; Phin, *Phytophthora infestans*; Phrm, *Phytophthora ramorum*; Phsj, *Phytophthora sojae*; Hpa, *Hyaloperonospora arabidopsidis*; Thps, *Thalassiosira pseudonana*; Phtr, *Phaeodactylum tricornutum*.
(XLS)

Table S4 **Whole transcriptome sequencing data for four** ***Pythium*** **species.** Sequence reads from each species were mapped to the respective genome using TopHat v1.1.4 [105]. Fragments per kilobase pair of exon model per million fragments mapped (FPKM) values were calculated using Cufflinks v0.9.3 [107] and genome annotation. Genes are considered expressed if FPKM value and FPKM 95% confidence interval lower boundary was greater than 0.001 and zero, respectively.
(XLS)

Table S5 **Protein domains enriched or depleted in core** ***Pythium*** **and** ***Pythium*** **species-specific clusters.** Fold change numbers are color coded as black for enriched and red for depleted domains. Only protein domains significantly enriched or depleted are shown.
(XLS)

Table S6 **Sizes of carbohydrate-active enzyme (CAZyme) classes in the stramenopile genomes.** The CAZymes coding genes were automatically annotated using the CAZymes Analysis Toolkit – CAT [62] according to the CAZy [61] database classification in combination with protein family domain assignment. Bold text indicate total. Pap, *Pythium aphanidermatum*; Par, *Pythium arrhenomanes*; Pir, *Pythium irregulare*; Piw, *Pythium iwayamai*; Puls, *Pythium ultimum* var. *sporangiiferum*; Pult, *Pythium ultimum* var. *ultimum*; Pve, *Pythium vexans*; Phin, *Phytophthora infestans*; Phrm, *Phytophthora ramorum*; Phsj, *Phytophthora sojae*; Hpa, *Hyaloperonospora arabidopsidis*; Thps, *Thalassiosira pseudonana*; Phtr, *Phaeodactylum tricornutum*.
(XLS)

Table S7 **Quantitative comparison of pathogenicity-related proteins in stramenopiles.** Genes were predicted for all datasets using PFAM v23.0 prediction and BLASTP searches. Results were compared to published genome datasets for *Pythium ultimum* var. *ultimum* [53], *Phytophthora* species [6], [7], *Hyaloperonospora arabidopsidis* [52] and *Thalassiosira pseudonana* [58]. The numbers in the denominator show the number of genes with expression support. Genes are considered expressed if FPKM value and FPKM 95% confidence interval lower boundary was greater than 0.001 and zero respectively. The expression data for *Pythium ultimum* var. *ultimum* were taken from Lévesque *et al.* [53]. Pap, *Pythium aphanidermatum*; Par, *Pythium arrhemonanes*; Pir, *Pythium irregulare*; Piw, *Pythium iwayamai*; Puls, *Pythium ultimum* var. *sporangiiferum*; Pult, *Pythium ultimum* var. *ultimum*; Pve, *Pythium vexans*; Phin, *Phytophthora infestans*; Phrm, *Phytophthora ramorum*; Phsj, *Phytophthora sojae*; Hpa, *Hyaloperonospora arabidopsidis*; Thps, *Thalassiosira pseudonana*.
(XLS)

Table S8 **Number and percentage of secreted proteins from stramenopiles.** The table shows the number of secreted protein and the percentage of proteins to be secreted from 13 stramenopiles. Also shown is the number and percentage of secreted proteins with transcript support. The secreted proteins were identified by SignalP v3.0 [64] and transmembrane domains were predicted with TMHMM [65]. Protein coding genes are considered expressed if the FPKM value and FPKM 95% confidence interval lower boundary was greater than 0.001 and zero, respectively. The expression data for *Pythium ultimum* var. *ultimum* were taken from Lévesque *et al.* [53].
(XLS)

Table S9 **Gene ontology (GO) molecular function and biological process categories enriched in core** ***Pythium*** **and species-specific secretome.** The table shows the enrichment fold in core *Pythium* and specific-specific secretome as compared to the non-secretome. Only GO terms significantly enriched in secretome are shown.
(XLS)

Table S10 **Protein domains enriched in core** ***Pythium*** **and species-specific secretome.** The table shows the enrichment fold in core *Pythium* and species-specific secretome as compared to the non-secretome. Only domains significantly enriched in secretome are shown.
(XLS)

Table S11 **Protein domains enriched or depleted in stramenopile-core, diatom, Hpa and** ***Pythium*** **/** ***Phytophthora*** **-specific gene families.** The table shows the enriched or depleted protein domains in taxa-specific and stramenopile-core gene families as compared to rest of the genomes. Fold change numbers are color coded as black for enriched and red for depleted domains. Only Protein domains significantly enriched or depleted are shown. Hpa, *Hyaloperonospora arabidopsidis*.
(XLS)

Table S12 **Number of genes syntenic to** ***Pythium ultimum*** **var.** ***ultimum*** **.** Syntenic genes were identified through reciprocal best matches between gene models and block identification using MCscan [88].
(XLS)
