# Supplementary figures and images for: Comparative Genomics Reveals Insight into Virulence Strategies of Plant Pathogenic Oomycetes
Source: PLoS One. 2013 Oct 4;8(10):e75072. doi: 10.1371/journal.pone.0075072 (PMC3790786; doi:10.1371/journal.pone.0075072)

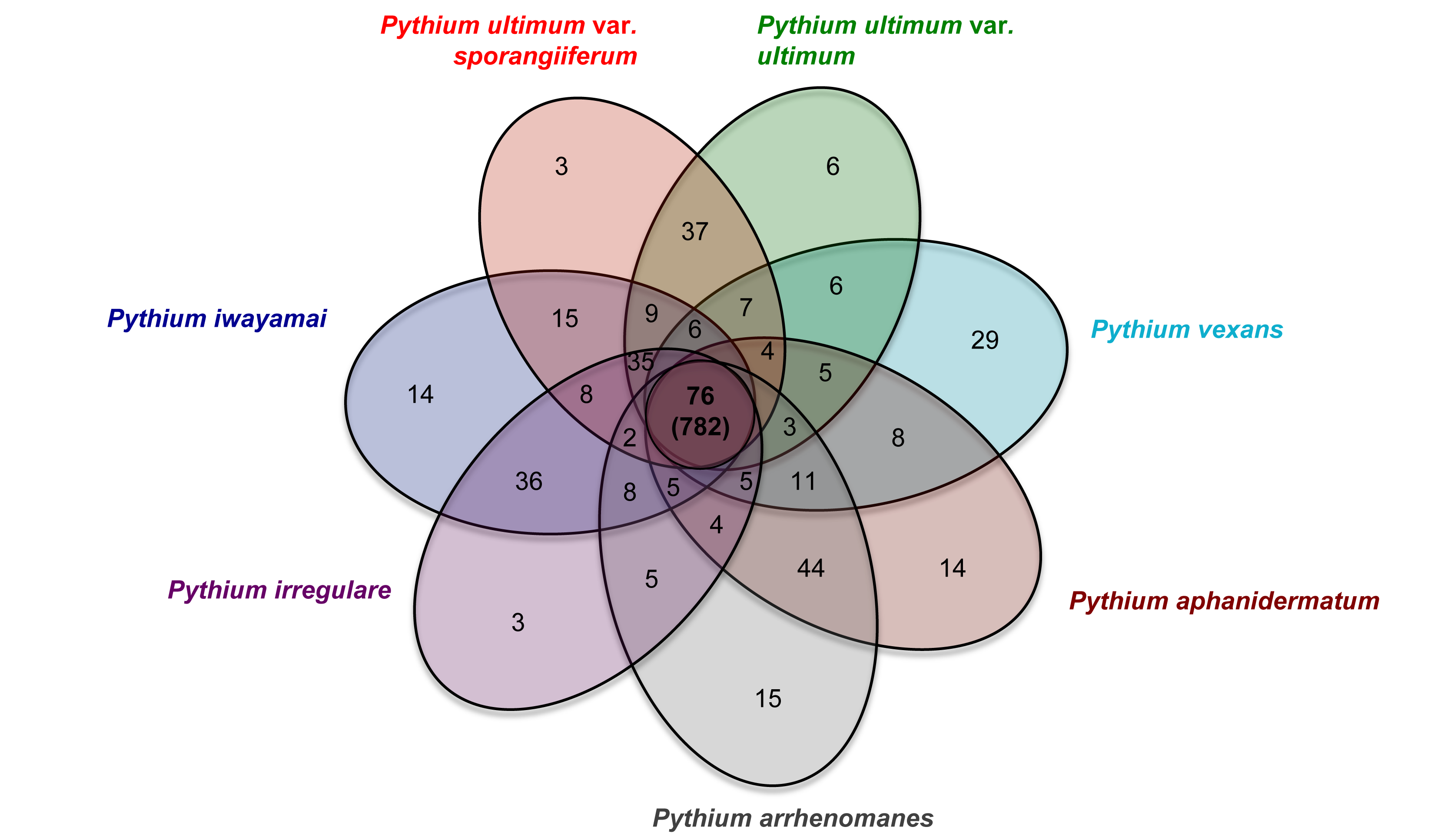

Supplement: Figure S1 — Shared clusters of secreted proteins in Pythium . The Venn diagram shows the distribution of secreted protein clusters among Pythium species. The putative secreted proteins from seven Pythium species were predicted by using SignalP v3.0 [64] and clustered using OrthoMCL [59]. The number of gene families (clusters) and the total number of clustered genes (numbers in parentheses) are indicated. (TIF) [file pone.0075072.s001.tif]
